# Comparing AI and human decision-making mechanisms in daily collaborative experiments

**DOI:** 10.1016/j.isci.2025.112711

**Published:** 2025-05-21

**Authors:** Linghao Wang, Zheyuan Jiang, Chenke Hu, Jun Zhao, Zheng Zhu, Xiqun Chen, Ziyi Wang, Tianming Liu, Guibing He, Yafeng Yin, Der-Horng Lee

**Affiliations:** 1 Institute of Intelligent Transportation Systems, College of Civil Engineering and Architecture, Zhejiang University, Hangzhou, China; 2 Institute of Intelligent Transportation Systems & Polytechnic Institute, College of Civil Engineering and Architecture, Zhejiang University, Hangzhou, China; 3 Department of Civil and Architectural Engineering and Mechanics, The University of Arizona, Tucson, AZ, USA; 4 Zhejiang Tranalytic Technology Company, Wenzhou, China; 5 Department of Civil and Environmental Engineering, University of Michigan, Ann Arbor, MI, USA; 6 Department of Psychology and Behavioral Sciences, Zhejiang University, Hangzhou, China; 7 Zhejiang University–University of Illinois Urbana Champaign Institute, Zhejiang University, Hangzhou, China

**Keywords:** Artificial intelligence applications, Computing methodology, Social sciences

## Abstract

Artificial intelligence (AI) is trying to catch up with human beings in many aspects. In this track, the potential for replacing human decision-making with AI models, such as large language models (LLMs), has become a topic of considerable debate. To test the performance of AI in daily decision-making, we compared humans, LLMs, and reinforcement learning (RL) in a multi-day commute decision-making game. It denotes a collaborative decision-making process where individual and collective outcomes are interdependent. We examined various performance metrics, including overall system results, system convergence progress, individual decision dynamics, and individual decision mechanisms. We find that LLMs exhibit human-like abilities to learn from historical experience and achieve convergence when making daily commute decisions. However, in the context of multi-person collaboration, LLMs still face challenges, such as weak perception of others’ choices, poor group decision-making mechanisms, and a lack of physical knowledge.

## Highlights

• LLMs learn human-like strategies in daily commute decisions but underperform humans/RL
• GPT-3.5 exhibits excessive stability; GPT-4 shows excessive decision volatility
• LLMs struggle with collaborative dynamics and physical knowledge comprehension
• RL aligns closely with human benchmarks, providing superior system efficiency

## Graphical abstract

**Figure:**
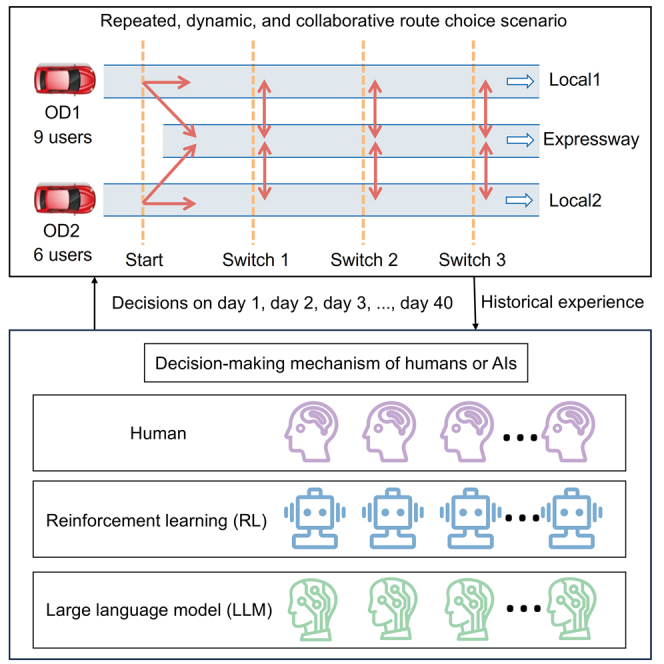

## Introduction

In contemporary society, individuals frequently make decisions within a framework of interdependence with their groups. The complexity of these decisions is primarily evident in how individual choices can influence group outcomes, which in turn affect future individual decisions. This analytical framework is extensively applied in areas, such as public goods dilemmas,^1^ stock trading,^2^ and transportation choice.^3^ Artificial intelligence (AI) is advancing swiftly toward human-level capabilities, especially in complex decision-making and inference tasks.^4^^,^^5^^,^^6^^,^^7^^,^^8^ With the advent of large language models (LLMs), the realization of artificial general intelligence (AGI) has become increasingly feasible, leading to potential implications for fields requiring interactive decision-making.^9^^,^^10^^,^^11^^,^^12^ The prospect of employing LLMs to model human social interactions and decision processes is promising, potentially enabling simulations of cooperative and competitive behaviors across diverse scenarios.^13^^,^^14^ However, the existing research presumes that LLMs mirror human cognitive mechanisms,^15^^,^^16^^,^^17^^,^^18^ but there is insufficient evidence that their decision-making processes align with those of humans, particularly in cases with collaborative decisions among multiple subjects. Significant limitations remain in LLM-based simulations of human decision-making. This prompts the question: Can LLMs effectively emulate or even surpass human decision-making capability in various social contexts, particularly in daily-life scenarios involving repeat, dynamic and collaborative decisions among people, such as commuting route choice? This gap is evident in collaborative and repetitive environments, which differs from the controlled environments of classical game theory studies. Previous studies have primarily focused on two key aspects of LLMs in game theory. The first is the challenges faced by LLMs in classical game theory: in these scenarios, two players make decisions simultaneously, and the outcomes of their interactions are expressed through a finite payoff matrix. Research has indicated that in 2 × 2 matrix games, LLMs still struggle to consistently select optimal strategies.^19^^,^^20^ Additionally, some researchers have proposed using larger-scale benchmarks to evaluate LLMs’ performance across various game scenarios in order to comprehensively analyze their strengths and weaknesses.^21^^,^^22^ At the same time, some studies have attempted to extend evaluations to more complex game scenarios, which often involve longer textual interactions. Results show that LLMs can exhibit certain strategic behaviors in communication-centric strategy games. For instance, in games characterized by deception and negotiation (such as Werewolf and Avalon^23^^,^^24^^,^^25^), LLMs demonstrate human-like strategic thinking, including deception, trust-building, and leadership abilities. However, existing research still suffers from several limitations. First, these experiments are mostly based on non-realistic scenarios, while classical game theory is merely a highly simplified abstraction of reality, characterized by static conditions, controlled environments, and one-shot decision-making. In the realm of strategy games, although the evaluated scenarios are more complex, they lack grounding in real-world contexts.

**Figure 1:**
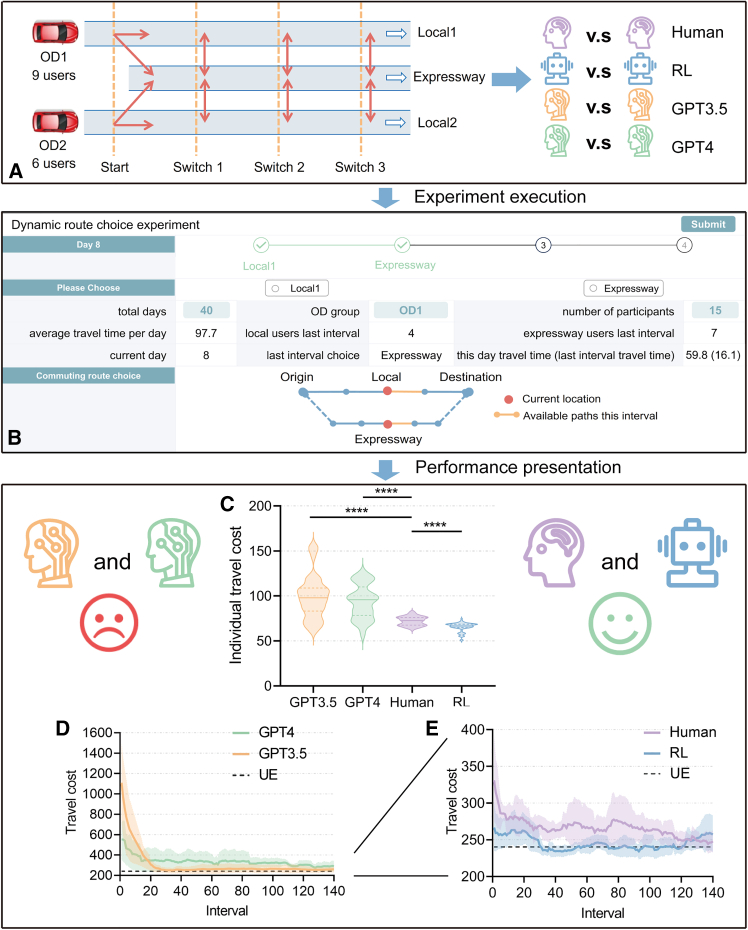
Overview of experimental procedures and summary of travel costs (A) Design of experiment. (B) This page is provided for experimental participants and includes information such as the number of users on local roads and expressways, among other details. (C) Individual users’ travel cost distribution, with lines indicating the median, 25th, and 75th percentiles. The statistical analysis was performed using the Kruskal-Wallis test, and the significance levels are represented as “ns” for *p* > 0.05, ∗*p* < 0.05, ∗∗*p* < 0.01, ∗∗∗*p* < 0.001, and ∗∗∗∗*p* < 0.0001. A smiley face signifies superior system performance, characterized by both a lower mean and variance, for both human and RL models. Conversely, a sad face denotes inferior performance, marked by higher mean and variance, for GPT-3.5 and GPT-4. (D) Convergence graph of total travel cost for GPT-3.5 and GPT-4, where each data point represents the 20-interval moving average. The solid line represents the average for five trails (75 participants), while the shaded area indicates the range between the 5th and 95th percentiles. The UE represents the travel time when all individual users (in the same OD group) experience equal travel times. (E) Convergence graph of travel cost between humans and RL.

To systematically explore the capabilities and boundaries of LLM-based decision-making, we apply experimental economic tools to evaluate and compare decisions of LLMs (GPT-3.5 and GPT-4), human participants, and reinforcement learning (RL). The experiment is designed for an everyday life game with multi-day, dynamic, and repetitive commute route choice decisions of users and their underlying travel times. This experimental paradigm, extensively validated within the transportation engineering field,^26^^,^^27^^,^^28^^,^^29^ facilitates the analysis of mechanisms underlying multi-day repetitive human decision-making processes, representing general daily life collaborative scenarios. In detail, we simulate a 40-day commuting route choice experiment. It features a one-way network with two origin-destination (OD) pairs, 15 users (9/6 for OD1/OD2, choosing between Local1/Local2 and expressway), and each chooses a starting route with opportunities to switch routes thrice during the commute within each day (see Figures 1A and 1B, and 160 mouse clicks of participants form a trial). Expressways typically offer shorter baseline travel times but are more sensitive to variations caused by fluctuations in user demand. Conversely, local roads exhibit greater stability and longer baseline travel times. From a risk-benefit perspective, expressways can be characterized as high-risk, high-reward routes, whereas local roads represent low-risk, low-reward alternatives. In this experimental paradigm, the road network state is influenced by individual route choices, with subsequent decisions based on the prior network state. Theoretical analysis suggests that road networks tend to evolve toward a state of user equilibrium (UE), where travel times on locals and expressways equalize, ensuring no individual can reduce their travel time by choosing an alternative route.^30^ The status of UE and system optimum (SO) for the road network is detailed in Table 1. The design of two OD pairs advances simple games to complex games in daily life, better assessing the performance of LLMs in multi-agent interactions (within and between diverse groups). By comparing LLM participants, human participants, and RL participants across independent trials, we assess system overall results, system converging progress, individual route switch dynamics, and individual decision-making mechanisms.

**Table 1:** SO and UE status of the road network

	Local1	Expressway	Local2	System
User equilibrium (UE)	4 (15.92)	7 (16.11)	4 (15.92)	15 (16.00)
System optimum (SO)	5 (17.25)	5 (7.89)	5 (17.25)	15 (14.13)

Our results and analyses gained insights applicable to scenarios involving repeated multi-person interactions. (1) Facing multi-day commuting decisions, LLMs can learn from historical experiences in a human-like manner, achieving convergence in the overall system state. (2) In collaborative multi-agent scenarios, there is still room for improvement in the perception and decision-making capabilities of LLMs. Specifically, LLMs require further optimization in perceiving others’ choices, optimizing group decision-making mechanisms, and improving physical knowledge. The conclusions derived from this study not only hold significant practical value in the field of urban traffic domain but, more importantly, establish a universal AI decision-assistance framework within dynamic and collaborative systems. In the future, the scope of our research can be extended to encompass applications in personal assistance and multi-agent social simulations. Within the domain of personal assistance, LLMs could be utilized to facilitate tasks such as orchestrating restaurant reservations or optimizing shopping decisions. Additionally, multi-agent simulations offer a promising avenue for investigating complex interactions, such as the influence of information dissemination within social networks on user emotions and subsequent behaviors.^31^ Furthermore, we could explore collaborative decision-making dynamics in financial markets, simulating stock trading behaviors driven by multi-agent LLM-based systems.^2^

## Results

### System overall results

The term “system overall results” primarily refers to outcomes derived from all user interactions within the system, emphasizing a static concept. An analysis of variance (ANOVA) revealed a significant main effect for the condition ($F_{\left(3,3196\right)}$ = 38.58, p < 0.0001). First, in terms of total travel time, no significant difference was observed between the RL ($M_{RL}$ = 245.75, SD = 49.94) and human ($M_{Human}$ = 270.43, SD = 107.34; p = 0.2410; 95% confidence interval (CI): −9.23, 58.59). For the GPT-3.5 model ($M_{GPT3.5}$ = 369.85, SD = 412.14), travel costs were significantly higher compared to the human trials (p < 0.0001; 95% CI, −133.3, −65.52). Similarly, the GPT-4 model ($M_{GPT4}$ = 339.15, SD = 307.59) also incurred higher travel time than human (p < 0.0001; 95% CI; −102.60, −34.81). This suggests that the traffic efficiency resulting from LLMs is inferior to that of humans and RL.

Next, we consider an individual’s travel cost. We employed the Kruskal-Wallis test to assess the statistical significance (Figure 1C). The results indicated that the travel costs of humans ($M_{Human}$ = 72.11, SD = 5.01) were significantly higher than those managed by RL algorithms ($M_{RL}$ = 65.53, SD = 4.89; p < 0.0001). In contrast, the travel costs for GPT-3.5 ($M_{GPT3.5}$ = 98.63, SD = 23.48) and GPT-4 ($M_{GPT4}$ = 93.58, SD = 18.82) were significantly higher than human (p < 0.001). The distribution of travel costs for RL trials was the most concentrated, followed by human trials. In comparison, the distributions for GPT-3.5 and GPT-4 were less concentrated, indicating greater variability in individual travel costs. According to the state of the UE, all participants should have the same travel time and should be treated equally. This suggests that LLMs may introduce substantial unfairness affecting individual users adversely. The concentration of travel costs in RL is due to the gradual decrease in the learning rate over time, which leads to stable decision-making upon convergence.

As shown in Figures 1D and 1E, the analysis of the convergence trend shows a decrease over time across all trials. This observation underscores that both AI (LLMs and RL) and human assimilate knowledge from historical experiences, which they subsequently utilize to inform their decision-making processes. Moreover, compared to LLMs, humans incur lower initial travel costs and smaller learning space to improve system travel efficiency. This phenomenon is attributed to humans possessing more robust prior knowledge, which facilitates rapid comprehension of the efficacy of current scenarios and enhances their decision-making processes.

Subsequently, we examined the travel costs associated with two OD pairs. The travel costs associated with OD1 were significantly higher than those for OD2 among humans, GPT 3.5, and GPT 4 (Table S1; Figure S1). Conversely, for RL trials, the difference in travel times between OD1 and OD2 was not statistically significant. In scenarios with uneven traffic demand (9 for OD1, 6 for OD2), both human and RL can effectively distribute traffic flow. In contrast, LLMs struggle to allocate traffic efficiently, leading to unfairness between OD pairs.

### System converging progress

**Figure 2:**
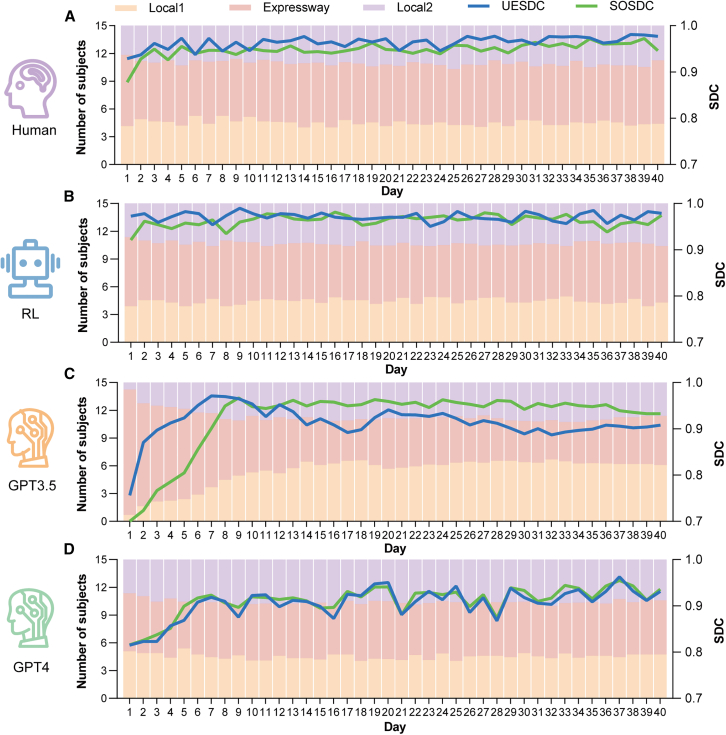
SDC coefficient and the number of users on different roads (A) Human trials. The line graph represents the SDC coefficient, while the bar chart represents the number of users on different routes. The value of the data is the average obtained after four decisions made throughout the day. (B) RL trials. (C) GPT-3.5 trials. (D) GPT-4 trials.

“System converging progress” refers to the dynamic state evolution of all users during interactive processes. In the economic evaluation of transportation systems, UE and SO serve as critical metrics for efficiency assessment. UE and SO are achieved by balanced and collaborative route choices among users: under UE, users could not further decrease their travel cost by route switches, indicating identical travel time for users of the same OD; whereas SO is characterized by minimized system costs. To quantify the similarity between the observed route choice behavior and UE or SO, we employ the Sørensen-Dice coefficient (SDC),^32^ which measures the resemblance between experimental and theoretical route choices. An SDC value closer to 1 indicates a higher similarity to UE (or SO). As shown in Figure 2, the results reveal that RL closely mirrors human benchmarks across multiple metrics, whereas GPT-3.5 and GPT-4 exhibit notable deviations. First, we conducted an ANOVA to test the user equilibrium Sørensen-Dice coefficient (UESDC) metric over the last 40 intervals. The results indicated no significant difference between humans ($M_{Human}$ = 0.973, SD = 0.034) and RL ($M_{RL}$ = 0.971, SD = 0.035; p = 0.996; 95% CI; −0.014, 0.017). However, humans showed significantly better convergence in UE compared to GPT-3.5 ($M_{GPT3.5}$ = 0.900, SD = 0.065; p < 0.0001; 95% CI: 0.057, 0.088) and GPT-4 ($M_{GPT4}$ = 0.925, SD = 0.090; p < 0.0001; 95% CI; 0.032, 0.064). For the system optimum Sørensen-Dice coefficient (SOSDC) metric, there was no significant difference between humans ($M_{Human}$ = 0.961, SD = 0.037) and the RL model ($M_{RL}$ = 0.962, SD = 0.036; p = 0.997; 95% CI: −0.013, 0.011). However, humans demonstrated significantly better convergence compared to GPT-3.5 ($M_{GPT3.5}$ = 0.945, SD = 0.037; p < 0.0001; 95% CI; 0.003, 0.028) and GPT-4 ($M_{GPT4}$ = 0.931, SD = 0.070; p < 0.0001; 95% CI; 0.017, 0.041). Vertically analyzing, GPT-3.5 showed fluctuations in UESDC but an increasing alignment with SOSDC. GPT-4, however, indicated a progressive alignment toward both UESDC and SOSDC, suggesting gradual improvements in collaborative decision-making. For humans, we observed optimizations of a relatively small magnitude. Nevertheless, we observed a consistent discrepancy in all experiments compared to UE. This finding corroborates the conclusion that UE is not fully attainable in route choice experiments, a principle that extends to AI implementations, as evidenced by the inability of all participants in the experiment to achieve equilibrium or maintain their choices without shifts.^3^^,^^33^ Deviations from UE are primarily caused by the unpredictability of individual decisions.

Additionally, we explored the number of users across different road segments to assess the system’s convergence process of route choices. We observed that throughout the experiment, GPT-3.5 demonstrated significant changes in the distribution of participants over roadways. Specifically, at the experiment’s onset, most participants initially choosing expressway shifted toward Local1 and Local2. We attribute this to a notable learning process in GPT-3.5, where the initial convergence of most participants led to significant congestion, prompting a redistribution of route choices in the later stages of the experiment. In contrast, the number of participants in all other trials remained stable, indicating that convergence in the distribution of road segments was achieved.

### Individual route switch dynamics

**Figure 3:**
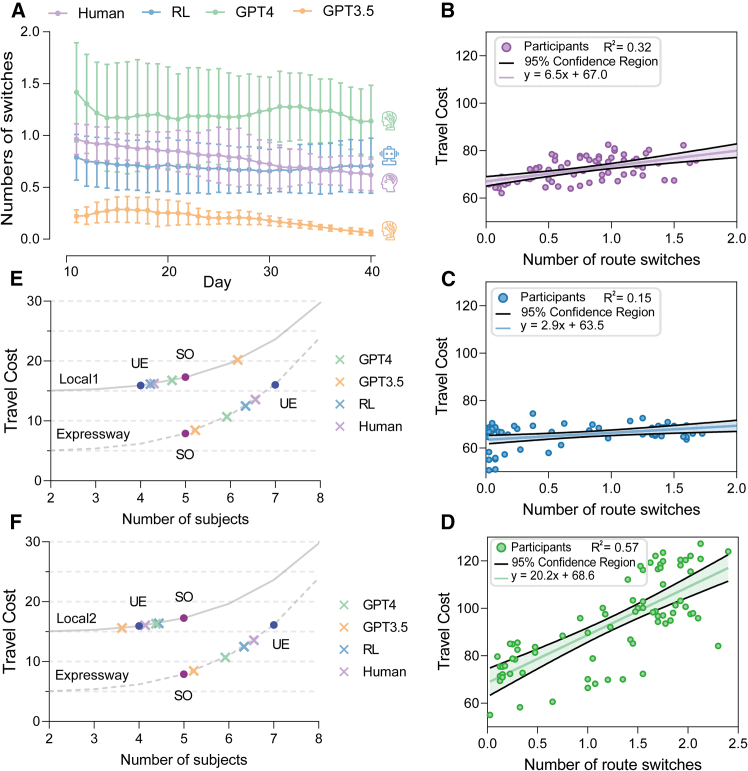
Dynamic outcomes of the commuting network (A) Convergence graphs of the number of route switches per day over time for all trials, where each data point represents a 10-day moving average. The error bars represent the standard deviation. (B) Linear regression of human travel costs versus the number of switches, with each data point representing the value for each participant. The black solid line indicates the 95% confidence region. (C) Regression curve for RL. (D) Regression curve for GPT-4. (E) Average number of OD1 participants on Local1 and expressway during the last 10 intervals, with UE and SO points. (F) Average number of OD2 participants on Local2 and expressway.

In this study, the term “route switch” refers to participants choosing a different path in the current interval from the previous interval. The frequency of route switches assessed the traffic dynamics of road network states. We conducted an ANOVA to analyze whether there were differences in route switches frequency among different groups. The ANOVA revealed a significant main effect ($F_{\left(3,796\right)}$ = 341.5, p < 0.0001), indicating substantial variability in route-switching behavior across groups. When comparing the average daily route switching frequency between the RL and human participants, no significant differences were observed ($M_{RL}$ = 0.71, SD = 0.27 vs. $M_{Human}$ = 0.78, SD = 0.29; p = 0.156; 95% CI: −0.016, 0.156). The GPT-4 model exhibited a higher switching frequency compared to human (($M_{GPT4}$ = 1.24, SD = 0.52; *p* < 0.0001; 95% CI: −0.541, −0.369). In contrast, the GPT-3.5 model demonstrated a significantly lower frequency of route switches ($M_{GPT3.5}$ = 0.17, SD = 0.15) compared to human participants (p < 0.0001; 95% CI: 0.526, 0.698). The analysis indicated that, among all trials compared, the traffic stability was highest in GPT3.5, whereas GPT4 exhibited the greatest instability. From a dynamic perspective, Figure 3A illustrates that both humans and GPT-3.5 exhibit a trend of gradually stabilizing in their average daily route switching frequency over time, indicating individual learning and adaptation. In contrast, RL remained stable throughout, while GPT-4 fluctuated significantly without stabilizing. To investigate the network stability caused by individual route switches, the relationship between the average travel cost and the number of route switches was explored through correlation analysis across all trials. Prior research indicates that an increase in the frequency of route switching correlates with longer travel times for individuals.^34^ As depicted in Figures 3B–3D, there is a positive correlation between travel cost and route switches (Human: $R^{2}$ = 0.32, p < 0.0001; RL: $R^{2}$ = 0.15, p = 0.0014; GPT-4: $R^{2}$ = 0.57, p < 0.0001). The correlation analysis showed no significant relationship between travel cost and route switching in GPT-3.5 ($R^{2}$ = 0.01, p = 0.38). Previous research indicates that a greater regression slope is positively correlated with increased instability within the road network system.^35^ Analysis of these slopes reveals that RL achieves the highest stability, human participants display moderate stability, while GPT-4 shows considerable instability. Notably, the decision-making framework of GPT-3.5 diverges markedly from human strategies, suggesting distinct underlying mechanisms influencing stability.

We analyzed passenger distribution across two local routes and expressways in comparison with UE and SO performance metrics. In this study, expressways, characterized by higher risks and opportunities, contrast with local routes, which present lower risks and opportunities. By comparing the deviation from the UE averages, we can infer individual risk preferences. Detailed results are illustrated in Figures 3E and 3F. The analysis indicated that traffic volumes on expressways were consistently below the UE state, whereas for local routes (Local1 and Local2), volumes were predominantly above UE, except for the GPT-3.5 model on Local2. This discrepancy was attributed to a risk avoidance inherent in the traffic model.^35^^,^^36^ Specifically, when expressway volumes exceed those of the UE, a substantial penalty is incurred, prompting users to prefer the local to mitigate risk. The results showed that LLMs were more risk-averse compared to humans, likely due to perceiving greater uncertainty in road commuting systems. This uncertainty, enhanced by a congestion aggregation effect, led to significant initial travel delays. This observation is corroborated by the initial travel time illustrated in Figure 1D. LLMs also showed higher variance in route choice, indicating less stability (Figure S2). This variability tended to make LLMs prefer more reliable routes under uncertain conditions.

### Individual decision-making mechanism

**Figure 4:**
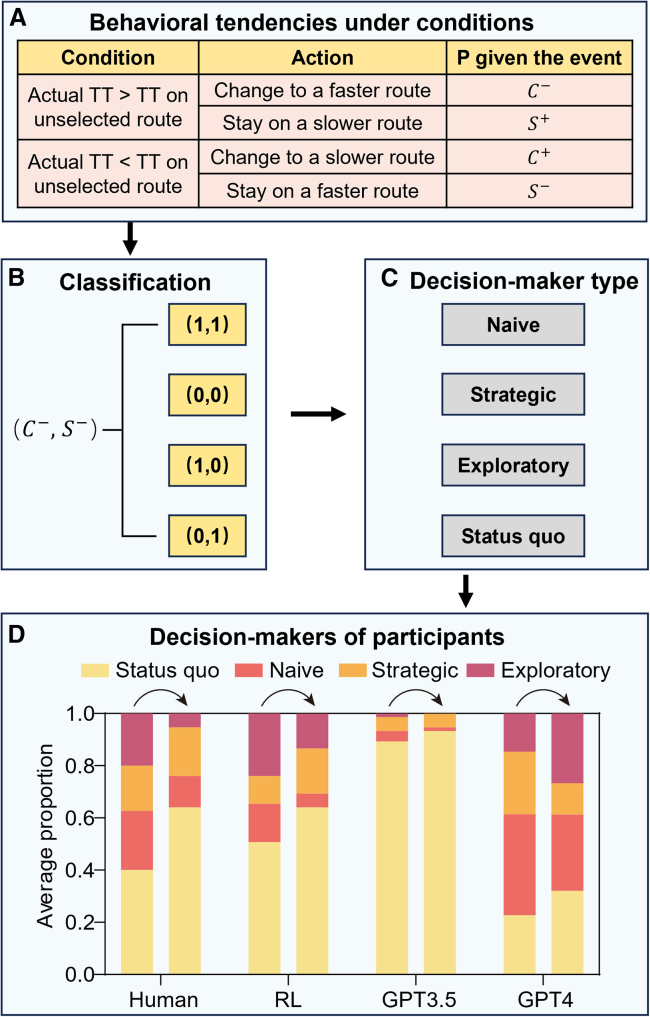
Theoretical framework and empirical outcomes of user classification (A) Definition of conditional probability for different behaviors. (B) User classification based on two-dimensional vectors. (C) Four distinct user types. (D) Classification results of four kinds of participants. The first column represents the types of users during the initial 40 intervals (days 1–10), and the second column shows user types in the final 40 intervals (days 31–40).

To elucidate the distinctions between human and AI in repetitive decision-making processes, we integrated route switching to examine the differences in decision-making types among users. Specifically, we employed conditional probability to model the switching decisions across various states of the road network. We categorized each user into one of four distinct types, as shown in Figure 4. *Naive* decision-makers always choose the route with the perceived shortest travel time, reacting only to real-time information. *Strategic* decision-makers avoid routes that others are more likely to choose, which are intuitively selected due to their shorter travel time in the previous step, and instead opt for less congested alternatives. *Exploratory* decision-makers frequently switch routes without considering past traffic conditions. *Status quo* decision-makers stick to their initial choice regardless of changing traffic patterns.

The initial focus of this study was on human decision-maker mechanisms, where the proportions of *status quo*, *naive*, *strategic*, and *exploratory* decision-makers were initially at 40.0%, 22.7%, 17.3%, and 20.0%, respectively. In this situation, *status quo* decision-makers dominated, while the remaining types held moderate proportions. In contrast, RL exhibited a higher proportion of *status quo* decision-makers (50.7%). GPT-3.5 predominantly consisted of *status quo* decision-makers (89.3%), with minimal representation of other types (*Naive*: 4.0%; *Strategic*: 5.3%; *Exploratory*: 1.4%). This overly static decision type structure was likely to trap the group in suboptimal states, thereby hindering the attainment of UE. For GPT-4, there was a lower proportion of *status quo* decision-makers (22.6%), but a significantly higher proportion of *naive* decision-makers (38.7%). This composition led to excessive volatility and instability.

Further analysis of the transition in decision-making types from the start to the end of the experiment revealed that humans tend to shift toward *status quo* over time. The proportion of human *status quo* decision-makers increased from 40.0% to 64.0%, while *naive* and *exploratory* decision-makers decreased from 22.7% and 20.0% to 12.0% and 5.3%, respectively. This reflected experiential learning of equilibrium concepts, indicating that humans, after a period of exploration and accumulating sufficient historical experience, tended to choose what they perceived as the better decision.^34^^,^^35^ This highlighted the strong capacity of humans to learn and refine their decision strategies from experience. However, in the LLM experiment, due to the dominance of *status quo* decision-makers, GPT-3.5 showed little change, with *status quo* decision-makers increasing from 89.3% to 93.3% while *naive* decreased from 4% to 1.3%. Conversely, GPT-4 saw an increase in *status quo* decision-makers from 22.7% to 32%, while *exploratory* decision-makers rose from 14.7% to 26.7%. Combining these results with previous convergence outcomes, it can be inferred that GPT-4 also failed to learn satisfying strategies from historical experiences. These findings suggest that LLMs possess weaker experiential learning capabilities, undermining their collaborative performance.

## Discussion

In this study, we employed a well-established research paradigm from the field of transportation engineering, the multi-day repetitive commuting route choice experiment, to assess the decision-making capabilities of two types of LLMs and RL compared to human participants. This methodological approach enables an in-depth analysis of the multi-day decision-making processes essential for applying LLMs in human decision support. The results of our experiment lead to several key conclusions. Firstly, in terms of overall system travel time and stability status, LLMs can reduce travel time by learning from experience. However, they demonstrated inferior performance compared to human participants, indicating a significantly higher total travel cost compared with humans and RLs. Furthermore, while LLMs are generally capable of achieving convergence, their convergence process is notably slow, and the final state often deviates significantly from UE and SO. From an individual perspective, the distribution of travel costs among LLM participants was more uneven compared to humans, further highlighting issues of fairness. Regarding the frequency of route switching, GPT-3.5 participants showed lower frequencies, whereas GPT-4 participants exhibited higher frequencies. Additionally, LLM participants displayed a heightened sense of risk aversion compared to human participants. Examining the commuting system’s dynamics, LLMs have greater overall dynamism and instability for both within-day and day-by-day decisions. In terms of population composition, GPT-3.5 tended to consist of more decision-makers who maintained *status quo*, whereas GPT-4 included a higher proportion of *naive* decision-makers. In contrast, the population structure of RL closely mirrored that of human participants, suggesting a more human-like approach in decision-making dynamics.

Our experimental results indicate that LLMs exhibit human-like capabilities in making daily commuting decisions. Specifically, they can learn from historical experiences and achieve convergence in overall system performance. Analyzing travel costs and convergence metrics (SDC), we observe that LLMs are able to optimize gradually from unstable network states (far from UE) toward more favorable experimental conditions (closer to UE). Previous studies have demonstrated that LLMs possess reasoning and decision-making abilities similar to humans.^37^ Our research extends these findings to the transportation domain, suggesting that LLMs’ reasoning and decision-making capabilities remain effective in repeated daily travel decision-making scenarios. However, we identify several areas for improvement when LLMs operate in collaborative multi-agent environments. Firstly, the suboptimal traffic efficiency can be attributed to its lack of collaborative capabilities within multi-person settings. Specifically, in multi-person cooperative games, LLMs exhibit insufficient perceptual abilities to understand and predict the choices of other participants. Instead of adapting strategies that could lead to an optimal collective outcome (like human and RL), it persistently adheres to its preferred courses of action.^38^ The multi-OD scenario we adopted further simulates complex gaming scenarios in reality, making it more challenging for LLMs to collaborate with others. In the context of this experiment, this behavior manifested as significant congestion during initial stages. Even though it would have been intuitive to choose routes counter to the group’s trends to alleviate this congestion, the LLMs continued to favor its predetermined navigational preferences. This reasoning can be substantiated by the system’s overall results and converging progress. Specifically, in terms of travel costs within traffic systems, individual travel costs, and convergence metrics (SDC), LLMs consistently underperform humans.

Secondly, our research suggests that the suboptimal group game performance of LLMs may be attributed to imbalances in their decision-making type structures. Prior studies indicate that a balanced diversity among different user types can optimize system performance, as seen in traffic systems where a mix of direct and contrarian route choices helps maintain stability.^39^ In a hypothetical scenario where all users follow only preceding information, the simultaneous selection of the same route can lead to significant system instability and degradation. Specifically, GPT-3.5 tends to adopt conservative strategies due to an overly concentrated decision-making type, resulting in stable but suboptimal conditions. In contrast, GPT-4’s dynamic decision-making mechanism, characterized by an excessive reliance on naive decisions, reduces effectiveness due to a lack of balance in user decision types. Specifically, the imbalanced population structure may generate overly homogeneous decisions, potentially reducing overall system effectiveness. These insights underscore the importance of incorporating diverse decision-making frameworks within LLMs to enhance their applicability and performance in complex environments.

Thirdly, it is hypothesized that the limitations in the gaming capabilities of LLMs may be attributed to their lack of genuine physical world perception. In contrast to humans, who can retrieve and compare similar events from long-term memory to develop new concepts, LLMs operate solely on textual input, lacking the multimodal experience necessary to construct integrated world models.^40^^,^^41^ This hypothesis is supported by observing the convergence speed across different subjects, wherein humans exhibit superior performance on various metrics (travel time and degree of convergence) in the initial 20 decision intervals of experimentation. This superior human performance can be attributed to innate knowledge and experiential understanding, which enable more accurate assessments of current situations (travel time) and significantly mitigate risks (severe traffic congestion). In RL, the strong learning and game-playing capabilities, akin to human abilities, arise because we define RL’s states and rewards based on human understanding of the problem, which differs from the perception approach used in LLMs. However, it is important to note that the absence of pre-existing knowledge in LLMs does not preclude the capability to learn from experience; indeed, these models demonstrate a discernible trend toward decreasing total travel costs.

Given the observed deficiencies in LLMs’ multi-day repetitive decision-making and reality comprehension within gaming contexts, developing strategies to enhance these abilities is crucial. As LLMs’ capabilities in this area are enhanced, their potential as auxiliary tools for everyday human travel decisions becomes more feasible. In the field of transportation, LLMs have already demonstrated extensive applications in decision-making processes, such as in the domain of autonomous driving. LLMs can serve as the central decision-makers for autonomous driving systems.^42^ Furthermore, LLMs have the potential to revolutionize user interactions with transportation systems by offering personalized travel assistance through natural language interfaces.^43^ The preceding analysis suggests that the principles derived from our study may be applicable to a broader range of repeated complex decision-making over multiple days. In scenarios individual user decisions are significantly influenced by the collective decisions of others, and individuals find it difficult to discern the sources of competition.^44^^,^^45^^,^^46^ For instance, restaurant patrons must consider the popularity of a venue to avoid overcrowding, shoppers weigh the costs and benefits of popular products, and investors assess the impact of extreme enthusiasm on stock valuations. These situations highlight how chaotic group dynamics can influence individual decisions in complex decision-making scenarios. Future research would further explore the potential of LLMs in simulating human behavior, especially in the key areas of prompt engineering and conversational memory management. Frist, by designing precise prompt, we can enable LLMs to not only understand how group behaviors influence the system outcome but also predict other individual behavioral patterns. This capability is crucial for enhancing the interactivity of AIs and can help us better simulate and assist complex social dynamics. For example, in situations involving multi-agent dynamic and repeat interactions, an LLM should be able to identify subtle changes among participants and adjust its responses accordingly. Second, based on conversational memory management, LLMs can improve their ability to handle long-term information and uncover behavioral patterns or mechanisms of social interaction hidden beneath surface-level communication. This includes extracting useful patterns from large amounts of unstructured historical dialog text, automatically adjusting the depth of memory for specific topics based on context, and proactively recalling relevant information at appropriate moments to facilitate more natural communication.

As LLMs continue to advance and become more widely adopted, we may enter a new era of human-AI collaborative decision-making. This hybrid scenario is likely to become increasingly common across various fields. In this cutting-edge area, there are several social behavioral phenomena worth attention, such as people’s attitudes toward LLMs. On one hand, as individuals grow accustomed to working with AI, new types of social dynamics may emerge, such as “bullying behavior”. Such behaviors could stem from curiosity, distrust of the technology, or viewing AI as an entity that can be arbitrarily treated. This not only impacts the quality of interactions but also poses challenges to fostering a harmonious human-AI society. Besides, disparities in information acquisition can intensify information asymmetry, as LLMs may exploit their advanced resource-gathering capabilities, potentially creating inequities between human users and AI systems. In summary, LLMs hold significant potential as decision-support tools in various everyday contexts. By leveraging their capacity to process and analyze large datasets; with more research attention and efforts, LLMs can aid in formulating strategies that benefit individual decision-makers and contribute to the overall efficiency and stability of the systems.

### Limitations of the study

Although this study contributes to the understanding of comparative decision-making capabilities among LLMs, RL models, and human participants in multi-day commuting environments, several limitations warrant discussion. First, the experimental design relies on simplified assumptions and abstractions to simulate real-world commuting scenarios. While the multi-OD framework captures certain complexities inherent in transportation systems, it fails to account for dynamic real-world factors such as weather conditions, infrastructure constraints, and real-time traffic disruptions. These elements often exert a critical influence on commuting decisions in practical contexts. Consequently, the observed behaviors of LLMs may not be fully generalizable to real-world applications. Second, the scope of LLMs tested in this study is relatively narrow. While GPT-3.5 and GPT-4o serve as representative examples of contemporary LLMs, the rapidly evolving landscape of AI models includes emerging architectures that integrate multimodal capabilities, enhanced reasoning mechanisms, or hybrid frameworks. These next-generation models may employ fundamentally different decision-making strategies. By restricting the analysis to two LLMs, the study limits its applicability to the broader spectrum of AI systems. Besides, the study does not address the influence of external factors on decision-making dynamics, particularly in human participants. Unlike AI systems, human decision-making is shaped by prior knowledge, habitual behaviors, and external stimuli, such as social norms, collaborative incentives, or competitive pressures. These inherent cognitive and experiential disparities require a nuanced framework for comparison. Future research should incorporate external influences to better elucidate the divergence between human and AI-driven decision-making processes. Finally, we did not specifically analyze or control for the influence of gender factors on the experimental results. This limitation may restrict the applicability of the study’s conclusions to different gender groups, thereby affecting the generalizability of the results.

## Resource availability

### Lead contact

Further information and requests for resources should be directed to and will be fulfilled by the lead contact, Zheng Zhu (zhuzheng89@zju.edu.cn).

### Materials availability

This study did not generate new unique materials.

### Data and code availability

• The datasets generated and analyzed during this study are available in the zenodo Data: https://doi.org/10.5281/zenodo.15307283.
• All code used in this study has been deposited in zenodo Data: https://doi.org/10.5281/zenodo.15307283.
• Any additional information required to reanalyze the data reported in this paper is available from the lead contact upon request.

## Acknowledgments

The work described in this paper was partially supported by 10.13039/501100001809Natural Science Foundation of China (72401255 and 72350710798), Natural Science Foundation of Zhejiang pvovince, China (LZ25E080007), and the Smart Urban Future (SURF) Laboratory, Zhejiang Province.

## Author contributions

Conceptualization, L.W., J.Z., and Z.Z.; methodology, L.W., C.H., and Z.Z.; writing—original draft, L.W., Z.J., C.H., J.Z., and Z.Z.; writing—review and editing, L.W., Z.J., C.H., J.Z., Z.Z., X.C., Z.W., T.L., G.H., Y.Y., D.-H.L.; funding acquisition, Z.Z. and X.C.

## Declaration of interests

The authors declare no competing interests.

## STAR★Methods

### Key resources table

**Table:** 

REAGENT or RESOURCE	SOURCE	IDENTIFIER
**Software and algorithms**		

Python	Python Software Foundation	https://www.python.org
GraphPad Prism	GraphPad Software	https://www.graphpad.com/
GPT API transfer station	V3 API	https://api.gpt.ge/
All code used in this paper	This paper	https://doi.org/10.5281/zenodo.15307283

### Experimental model and study participant details

A total of 75 undergraduate and graduate students (38 females; mean age = 22 years; self-reported ethnicity: 100% Asian) from Zhejiang University were recruited to participate in this experiment. All participants were randomly assigned to different experimental groups to ensure group equivalence and to minimize selection bias. The recruitment criteria are as follows: (1) participants must be at least 18 years old; (2) participants must possess normal vision or corrected-to-normal vision; (3) participants must be in good physical health with no major illnesses or interference from medications; (4) participants must have no history of mental or neurological disorders; and (5) participants must be able to understand and comply with the requirements of the study. The experimental procedures were approved by the Research Ethics Committee of College of Biomedical Engineering & Instrument Science, Zhejiang University (Reference Number: Zhejiang University Biomedical Engineering Ethics Review [2024] No. 5). All participants provided written informed consent before the experiment, and the possible consequences of the studies were explained.

### Method details

#### Experimental design

We propose an experimental design to simulate urban expressway traffic dynamics over 40 days, focusing on the commuting route choice. As shown in Figure 1A, this design incorporates a one-way road network with two distinct OD pairs, 9 users for OD1 and 6 users for OD2. Each pair initiates travel from different local roads (OD1/OD2 for Local1/Local2) but converges onto a shared expressway (Expressway). Commuters confirm their initial route choice at departure each day, distributed across four intervals. During their commute, participants have the opportunity to switch routes three times via access ramps, without incurring ramp travel time. This experimental setup addresses limitations identified in previous route choice studies, primarily utilizing networks with two or three routes.^34^^,^^35^^,^^47^ These earlier models demonstrated limited capacity in replicating complex traffic dynamics and real-world conditions. By introducing a more intricate network with dynamic route-switching capabilities, our design aims to enhance the realism of the simulation and capture the adaptive behaviors of commuters more effectively.

The Bureau of Public Roads (BPR) function is employed to quantify the travel cost on each road according to the equation:

(Equation 1) $$\begin{array}{l} c_{j}\left(n_{j}\right)=a_{j}\cdot \left(1+\alpha {\left(\frac{n_{j}}{s_{j}}\right)}^{\beta }\right) \end{array}$$

Here, $c_{j}$ denotes the travel cost on route j, $n_{j}$ is the number of users, $a_{j}$ is the free flow travel time, and $s_{j}$ is the capacity of route j. Parameters α and β are constants specific to the model. The BPR function is widely utilized due to its empirical validation and its flexibility in reflecting different road attributes by adjusting these parameters.

For local roads (Local1 and Local2), the BPR function parameters are set as follows: $a_{j}=15,\alpha =0.15,s_{j}=5\text{,}$ and β=4. For the expressway, the parameters are: $a_{j}=5,\alpha =0.075,s_{j}=3\text{,}$ and β=4. Based on the parameters, it can be observed that expressways are relatively more sensitive roads. This design aligns with previous studies.^35^

These formulations indicate that although the expressway offers a lower free travel time, its susceptibility to congestion increases significantly as traffic volume grows. This characteristic suggests that the expressway represents a route where higher risks coexist with potential time-saving benefits.

Furthermore, the current status of UE and SO within this road network is shown in Table 1.

#### Participants

This study involved 75 human participants, RL algorithm, and two versions of GPT (GPT-3.5-turbo, GPT-4o) (5 trials for each kind of participants, *N* = 300). These GPT versions represented the pinnacle of model development at the time, exhibiting enhanced capabilities in reasoning, creativity, and contextual understanding compared to their predecessors.

For humans, the experiment was conducted in a computer laboratory equipped with multiple terminals, where 75 students were recruited and divided into five trials, each comprising 15 participants. Prior to the experiment, all participants signed an informed consent form and attended a 15-min tutorial that explained the game rules and the feedback information displayed on the screen (illustrated in Figure 1B). The main experiment lasted approximately 45 min, during which participants engaged in collaborative anonymous interactions via an online platform. After each round (decision interval), participants submitted their choices, and the server processed these inputs, calculated the outcomes, and prepared the information for the subsequent round. Notably, once the experimental information is processed, it is immediately returned to the human participants, who do not actually have to experience the traffic congestion they caused.

In terms of RL, the experiment involved creating an environment (env), within which 15 independent agents operated. These agents received the current state information and took action accordingly. Upon completion, the environment calculated the outcomes and updated the state and rewards for the next round.

In the context of LLMs, each user is treated as a distinct conversational instance. The design of experimental prompt encompasses a comprehensive task description and incorporates real-time informational feedback. The LLM models functioned independently with session-based memory only, lacking access to past or concurrent session data, and operating as separate experimental entities. The task description details the experimental background, including aspects of road network design and other relevant parameters. Real-time information consists of feedback from experimental data post-selection of all LLMs. The detailed prompt design is provided in the source code. The experimental procedure is structured as follows: Initially, 15 new dialogues are established, each devoid of prior memory, and the experimental context is introduced. Subsequently, each LLM selects an initial route and inputs the corresponding parameters into a collaborative environment. The environment processes these inputs, and the resulting observations are relayed to each LLM in real time. Upon receiving the updated observational data, each LLM adjusts and selects a new route based on this refreshed information. This iterative process continues throughout the duration of the 40-day experiment.

In this study, the primary objective is not to optimize the travel performance of LLMs, but rather to conduct a fair comparison between LLMs, RL algorithms, and human performance. Specifically, we aim to evaluate their behavior under standardized conditions, without relying on specially crafted prompts or prior domain-specific knowledge. To ensure fairness, we designed neutral and unbiased informational prompts for the LLMs, refraining from providing additional guidance or tailored information that could influence their performance.

In this context, RL is formulated as a Markov Decision Process (MDP). The definition is as follows:

**Agent**: In this study, an agent is conceptualized as a decision-maker engaged in route choices. The total number of agents corresponds to the number of participants in the experiment, each making independent route choice decisions based on real-time information.

**State**: The state is defined by the information available to agents during the decision-making process, encapsulated by the following parameters: $l_{t}$, the number of agents selecting the local at time (round or decision interval) t; $e_{t}$, the number of agents choosing the expressway at time t; $lc_{t}$, the travel time on the local at time t; $ec_{t}$, the travel time on the expressway at time t; $n_{t}$, the route chosen at time t−1 and t, the current time step. Thus, the state can be represented as:

(Equation 2) $$\begin{array}{l} s_{t}=\left[l_{t},e_{t},lc_{t},ec_{t},n_{t},t\right] \end{array}$$

**Reward**: The reward function is designed under the premise that agents are inherently selfish, aiming to minimize their individual travel time. Thus, the immediate reward $r_{t}$ at any time t is determined by the travel cost of the chosen route:

(Equation 3) $$\begin{array}{l} r_{t}=\left\{ \begin{array}{l} lc_{t}\;if\;a_{t}=local\\ ec_{t}\;if\;a_{t}=expressway \end{array}\right. \end{array}$$

The cumulative reward $R_{t}^{i}$ over a journey is then the sum of all individual travel costs:

(Equation 4) $$\begin{array}{l} R_{t}^{i}=\sum_{t=1}^{n}r_{t} \end{array}$$

**Action Space**: The action space comprises the route choices available to each agent. It is represented by a 2-dimensions discrete variable where $x_{1}$ indicates choosing the local and $x_{2}$ indicates selecting the expressway. The action at time t is denoted as:

(Equation 5) $$\begin{array}{l} a_{t}=\left[x_{1},x_{2}\right] \end{array}$$

The RL framework in this experiment mirrors the conditions of human experimental setups, with identical state spaces and reward structures. Each agent operates under a self-maximizing policy, solely optimizing personal outcomes without collaborative interactions. The IA2C algorithm is utilized to address challenges in RL. The hyperparameters can be found in Table S2. The RL design approach we adopt first involves pretraining the RL agent. During this stage, the agent learns foundational strategies and behavior patterns through extensive interactions with environments, thereby establishing a relatively stable and efficient decision-making framework. After completing pretraining, when carrying out specific tasks during actual deployment, we no longer perform real-time adjustments or updates to the neural network parameters of the agent.

#### Decision-making types

To elucidate the distinctions in the multi-day repetitive decision-making processes between humans and AI, we incorporate the route-switching probability to analyze the decision-making types among human and AI users in depth. Prior psychological studies have documented sequence dependence in repetitive or sequential decision-making scenarios, where outcomes from earlier choices influence subsequent decisions.^48^ In our commuting route choice experiment, it is reasonable to posit that the outcomes of prior decision intervals modulate the propensity for route switching in subsequent intervals.

The theoretical underpinning for classifying user decision-making mechanism is derived from the *win-stay, lose-shift strategy*^49^ and the concept of *cognitive hierarchy* used in game theory.^50^ This framework posits that individuals are predisposed to adopt strategies that have previously yielded favorable outcomes. This notion is further explored, demonstrating how individuals adapt their strategies based on outcomes in scenarios analogous to the Congestion Game.^34^^,^^51^ To further classify the decision-making mechanisms of users, conditional probability is employed to capture the behavior of route switching under various circumstances.^52^

In extending these concepts to route choice, a method of user classification is proposed, which employs both direct and inverse categorization based on users' historical choices and outcomes. The classification methodology is delineated in Figure 4. This approach utilizes conditional probabilities to quantify the likelihood of an individual adopting a particular strategy following either gain or loss from previous decisions. Figure 4A delineates these probabilities, offering a precise mathematical description of decision-making tendencies under specified conditions, encapsulated by the equation:

(Equation 6) $$\begin{array}{l} C^{-}+S^{+}=C^{+}+S^{-}=1 \end{array}$$

wherein $C^{-}+S^{+}$ describes cases with the condition "the chosen route in the previous interval has a longer travel time than the unchosen one" and $C^{+}+S^{-}$ describes the rest of cases with "the previously chosen route has a shorter travel time". The definitions are as follows: $C^{-}$ indicates switching to the route which has the shorter travel time in the previous interval, $C^{+}$ indicates switching to the longer travel time route, $S^{-}$ represents staying on the shorter travel time route, and $S^{+}$ represents staying on the longer travel time route. Here, ($C^{-}$, $S^{+}$) represent two-dimensional variables used to categorize users. Each decision-making type is characterized by users' responses to traffic information and prior experiences. Although users may experience changes in their decision-making mechanisms over the long term, we can assume that their decision-making mechanisms remain consistent in the short term. Specific details are as follows:

1. *Naive decision-makers*: These users exhibit a straightforward decision-making process by consistently opting for the route with a shorter travel time, irrespective of previous travel times. This behavior is represented by vector (1,1). *Naive* decision-makers respond directly to real-time information.
2. *Strategic decision-makers*: In contrast, *strategic* decision-makers anticipate the presence of *naive* decision-makers and deliberately choose longer routes, possibly to avoid congestion caused by the majority opting for shorter routes. This type is denoted by the vector (0,0), indicating a complete reversal in the decision-making approach relative to *naive* decision-makers.
3. *Exploratory decision-makers*: Characterized by their propensity to alternate routes frequently, *exploratory* decision-makers do not consistently account for traffic conditions from previous steps. This behavior is captured by the vector (1,0), signifying a type of route switching irrespective of previous traffic status.
4. *Status quo decision-makers*: These decision-makers prefer consistency by sticking to a chosen route regardless of the traffic conditions encountered. Represented by (0,1), these individuals maintain their initial route choices, demonstrating a resistance to change based on traffic information.

### Quantification and statistical analysis

Statistical analysis was performed using GraphPad Prism software. All bar graph data are presented as mean ± SEM. ANOVA was utilized to evaluate the overall travel cost, SDC index, frequency of route changes across various groups and the number of objects on different roads. The Kruskal-Wallis test was applied to assess travel costs among different individuals. The Mann Whitney test was utilized to access the travel cost for different OD pairs. A *p*-value of less than 0.05 was deemed significant. The significance levels are represented as "ns" for *p* > 0.05, "∗" for *p* < 0.05, "∗∗" for *p* < 0.01, "∗∗∗" for *p* < 0.001, and "∗∗∗∗" for *p* < 0.0001.
