# Supplementary material for: Comparing AI and human decision-making mechanisms in daily collaborative experiments
Source: iScience. 2025 May 21;28(6):112711. doi: 10.1016/j.isci.2025.112711 (PMC12167486; doi:10.1016/j.isci.2025.112711)
Supplement: Document S1. Figures S1–S3 and Tables S1 and S2 [file mmc1.pdf]

**iScience, Volume *28***

## Supplemental information

# Comparing AI and human decision-making mechanisms in daily collaborative experiments

**Linghao Wang, Zheyuan Jiang, Chenke Hu, Jun Zhao, Zheng Zhu, Xiqun Chen, Ziyi Wang, Tianming Liu, Guibing He, Yafeng Yin, and Der-Horng Lee**

**Figures: S1 to S3**

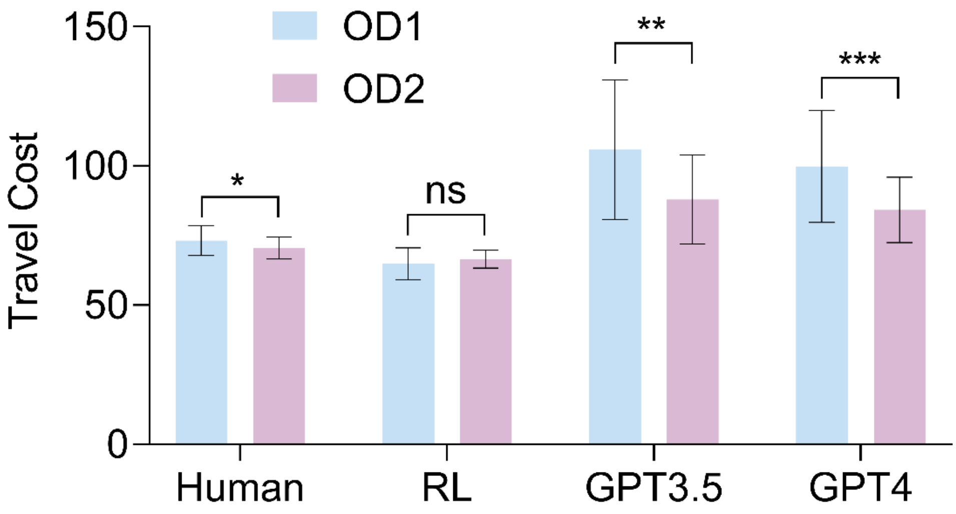


**Figure S1.** Travel time for different OD pairs. The statistical analysis was performed using the Mann Whitney test, and the significance levels are represented as "ns" for $p > 0.05$, "*" for $p < 0.05$, "**" for $p < 0.01$, "***" for $p < 0.001$, and "****" for $p < 0.0001$.

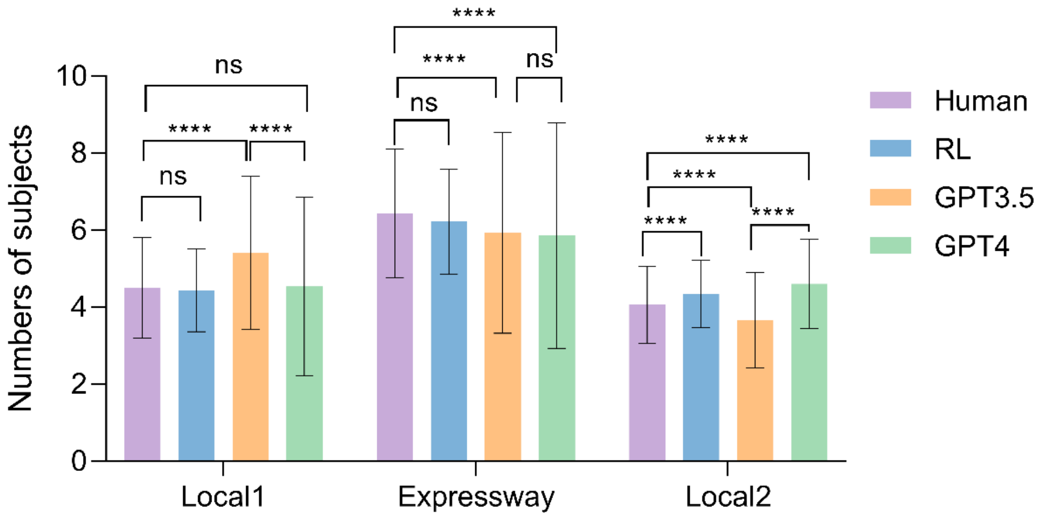


**Figure S2.** Numbers of subjects on different roads. The statistical analysis was performed using the ANOVA, and the significance levels are represented as "ns" for $p > 0.05$, "*" for $p < 0.05$, "**" for $p < 0.01$, "***" for $p < 0.001$, and "****" for $p < 0.0001$.

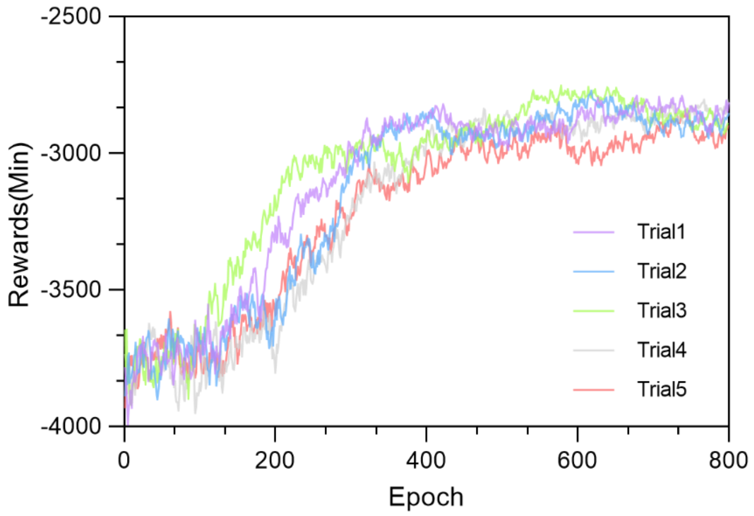


**Figure S3.** RL reward converging images.

## Tables: S1 to S2

**Table S1.** Mann Whitney test results for different OD pairs

| | Discovery? | P value | Mean rank of OD1 | Mean rank of OD2 | Mean rank diff. | Mann-Whitney U | q value |
|---|---|---|---|---|---|---|---|
| Human | No | 0.02636 | 42.56 | 31.17 | 11.39 | 470 | 0.01775 |
| RL | No | 0.55601 | 36.78 | 39.83 | -3.056 | 620 | 0.28079 |
| GPT3.5 | Yes | 0.00186 | 44.31 | 28.53 | 15.78 | 391 | 0.00188 |
| GPT4 | Yes | 0.00052 | 45 | 27.5 | 17.5 | 360 | 0.00105 |

**Table S2.** Hyperparameter setting of A2C

| Parameter | Value |
|---|---|
| Learning rate | 0.001 |
| Discount factor $\gamma$ | 0.99 |
| Parameter optimizer | 'Adam' |
| Entropy Coefficient | 0.01 |
| GAE | 0.01 |
| Training epoch | 1000 |
